# Modulation of Cell-Cycle Progression by Hydrogen Peroxide-Mediated Cross-Linking and Degradation of Cell-Adhesive Hydrogels

**DOI:** 10.3390/cells11050881

**Published:** 2022-03-03

**Authors:** Wildan Mubarok, Kelum Chamara Manoj Lakmal Elvitigala, Masaki Nakahata, Masaru Kojima, Shinji Sakai

**Affiliations:** Department of Materials Engineering Science, Graduate School of Engineering Science, Osaka University, Toyonaka 560-8531, Osaka, Japan; wildanmubarok@cheng.es.osaka-u.ac.jp (W.M.); kelum@cheng.es.osaka-u.ac.jp (K.C.M.L.E.); nakahata@cheng.es.osaka-u.ac.jp (M.N.); kojima@cheng.es.osaka-u.ac.jp (M.K.)

**Keywords:** gelatin, hyaluronic acid, Fucci2, horseradish peroxidase, degradation

## Abstract

The cell cycle is known to be regulated by features such as the mechanical properties of the surrounding environment and interaction of cells with the adhering substrates. Here, we investigated the possibility of regulating cell-cycle progression of the cells on gelatin/hyaluronic acid composite hydrogels obtained through hydrogen peroxide (H_2_O_2_)-mediated cross-linking and degradation of the polymers by varying the exposure time to H_2_O_2_ contained in the air. The stiffness of the hydrogel varied with the exposure time. Human cervical cancer cells (HeLa) and mouse mammary gland epithelial cells (NMuMG) expressing cell-cycle reporter Fucci2 showed the exposure-time-dependent different cell-cycle progressions on the hydrogels. Although HeLa/Fucci2 cells cultured on the soft hydrogel (Young’s modulus: 0.20 and 0.40 kPa) obtained through 15 min and 120 min of the H_2_O_2_ exposure showed a G2/M-phase arrest, NMuMG cells showed a G1-phase arrest. Additionally, the cell-cycle progression of NMuMG cells was not only governed by the hydrogel stiffness, but also by the low-molecular-weight HA resulting from H_2_O_2_-mediated degradation. These results indicate that H_2_O_2_-mediated cross-linking and degradation of gelatin/hyaluronic acid composite hydrogel could be used to control the cell adhesion and cell-cycle progression.

## 1. Introduction

The cell cycle is an intricate process for cell growth. Regulation of the cell cycle is fundamental to successful cell culture technology and tissue engineering. Additionally, dysregulation of the cell cycle is a hallmark of cancer cells, thus, cell-cycle modulation of cancer cells has been a target for developing anticancer drugs [1,2].

In the cellular microenvironment, physical cues including mechanical properties of the substrate and biochemical cues in the form of cell-adhesive ligands are known to modulate cellular behaviors including adhesion [3,4] and proliferation [5,6,7]. Gelatin and hyaluronic acid (HA) are abundantly found in various tissues of humans and animals [8,9,10], and are important biomaterials that facilitate strong biological activities [11,12,13]. The scaffolds composed of multiple polymers provide the microenvironment with multiple properties, attributed to each polymer. For example, gelatin/HA mixture could combine the cell-adhesive property of the arginine–glycine–aspartic acid (RGD) sequence and degradability of the gelatin with the physicochemical property and regulation of cell behavior and immune response function of HA. Therefore, various studies have reported the usefulness of gelatin/HA composite hydrogel scaffolds in tissue-engineering applications for biomimicking of the extracellular matrix (ECM) of the native tissues [10,14,15,16]. 

The biological activities of HA are related to its molecular weight. High-molecular weight HA is required for wound healing [17], while low-molecular-weight fragments of HA act as an activator of dendritic cells and macrophages during inflammation [18,19]. More importantly, the molecular weight of HA also governs oncogenesis: high-molecular-weight HA inhibits the progression of tumor growth, while low-molecular-weight HA has an opposing effect [20,21,22]. 

Hydrogen peroxide (H_2_O_2_) is widely used for degrading high-molecular-weight HA, gelatin, and various other polymers, through oxidative cleavage of the bonds [23,24,25,26]. Apart from polymer degradation, H_2_O_2_ is also used as an electron donor of horseradish peroxidase (HRP)-mediated reaction resulting in gelation of aqueous solutions of polymers with phenolic hydroxyl groups (Polymer–Ph), including Gelatin–Ph and HA–Ph. The hydrogelation through HRP-mediated cross-linking has been applied for a variety of biomedical applications, e.g., cell transplantation [27], bone regeneration [28], wound dressing [29], and inks for 3D bioprinting [30]. In the applications of using H_2_O_2_ for inducing HRP-mediated hydrogelation, no attention has been paid to the contradictory functions of H_2_O_2_ for inducing the degradation of the polymers. Recently, we reported that the contradictory function of H_2_O_2_ affects the mechanical property of hydrogels, which leads to changes in adhesion of the human adipose-derived stem cells (hASCs) and rat fibroblast cells [31]. However, the contradictory effect of H_2_O_2_ on other cell behaviors, such as cell-cycle progression, has not been investigated.

**Figure 1:**
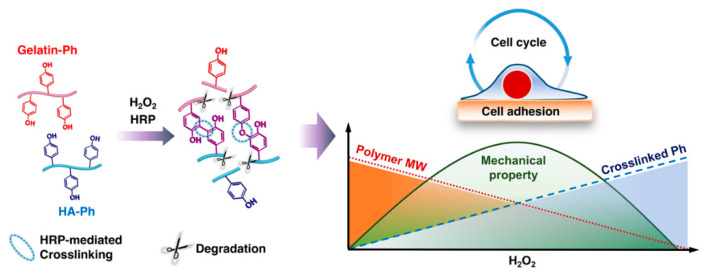
Schematic illustration of H_2_O_2_-mediated hydrogelation of Gelatin–Ph/hyaluronic acid (HA)–Ph composite through horseradish peroxidase (HRP)–mediated cross-linking of Ph moieties and polymer degradation, and its subsequent effect on cell adhesion and the cell cycle.

Cell-cycle modulation by the physical microenvironment of the cells has recently garnered great interest [32,33]. Although previous studies have reported the cell-cycle modulation by HA in collagen matrix [34], methacrylated gelatin [35], and chitosan–gelatin complex [36], no studies have investigated the cell-cycle modulation by Gelatin–Ph/HA–Ph composite hydrogel. This is particularly important considering that Gelatin and HA are known to be the component of the cancer microenvironment [33,37]. Therefore, in this study, we aimed to investigate the effect of H_2_O_2_ as an electron donor in HRP-mediated cross-linking and degradation-inducing oxidant of Gelatin–Ph/HA–Ph composite hydrogels on the cell-cycle progression (Figure 1).

In this study, we focused on the cell-cycle progression of cervical cancer cells, due to its high clinical prevalence worldwide, and being one of the highest causes of death in low-income countries [38]. Additionally, there is an effort to accelerate the cervical cancer elimination worldwide [39]. Hydrogel itself, including hyaluronic acid and gelatin, has been applied to tumor sites following tumor removal surgery to prevent tumor recurrence [40,41]. Here, we studied the cell-cycle progression of human cervical cancer cells using HeLa cells expressing genetically encoded fluorescent ubiquitylation-based cell-cycle indicator FucciHeLa cell is selected since it has been extensively used in biomedical study and research on cell-cycle regulation [42], while HeLa/Fucci2 cell has been used to study cell response following drug-induced cell-cycle arrest [43]. Fucci2 is composed of two chimeric fluorescent proteins, mCherry-hCdt1 and mVenus-hGem. Reciprocal accumulation of these two proteins turns cell nuclei red-fluorescent in G1 phase and green-fluorescent in the S/G2/M phases. 

Additionally, we also investigated the cell-cycle progression of non-transformed mouse mammary gland epithelial cell line (NMuMG) expressing FucciNMuMG is widely used to study the epithelial-to-mesenchymal transition (EMT), a physiological change that is necessary for cancer invasion and metastasis [44,45]. In addition, previous study has reported that HeLa/Fucci2 and NMuMG/Fucci2 have different cell-cycle response following chemical treatment [43]. Therefore, we studied both the HeLa/Fucci2 and NMuMG/Fucci2 cell cycle to investigate whether cell type-specific phenomenon also occurs when cultured on Gelatin–Ph/HA–Ph composite hydrogel with different physicochemical properties.

## 2. Materials and Methods

### 2.1. Materials

Gelatin (Type B; ca. 225 g bloom, bovine skin) was purchased from Sigma–Aldrich (St. Louis, MO, USA). Sodium hyaluronate (HA, MW ~1000 kDa) was purchased from Kewpie (Tokyo, Japan). Horseradish peroxidase (HRP, 140 U/mg), *N*-hydroxysuccinimide (NHS), *N*,*N*-dimethylformamide (DMF), hydrogen peroxide (H_2_O_2_) aqueous solution (31 *w*/*w*%), and catalase from bovine liver were purchased from Wako Pure Chemical Industries (Osaka, Japan). Water-soluble carbodiimide hydrochloride (WSCD·HCl) was obtained from Peptide Institute (Osaka, Japan). List of abbreviations used in this article is provided in Table 1.

### 2.2. Polymer–Ph Preparation

Gelatin–Ph (1.9 × 10^−4^ mol-Ph/g-Gelatin–Ph) and HA–Ph (1.5 × 10^−4^ mol-Ph/g-HA–Ph) were prepared by conjugating 3-(4-hydroxyphenyl) propionic acid with gelatin and tyramine hydrochloride with HA, respectively, using WSCD·HCl/NHS as previously reported [46,47]. 

### 2.3. Cell Culture

HeLa/Fucci2 and NMuMG/Fucci2 cell lines were obtained from RIKEN Bio-Resource Center (Ibaraki, Japan). HeLa/Fucci2 was cultured in Dulbecco’s Modified Eagle Medium (DMEM, Nissui, Tokyo, Japan) containing 10 *v*/*v*% fetal bovine serum (FBS). NMuMG/Fucci2 was cultured in DMEM supplied with 10 *v*/*v*% FBS and 10 μg/mL insulin (Sigma, St. Louis, MO, USA) [43]. Cells were cultured at 37 °C in a 5% CO_2_ incubator.

### 2.4. Hydrogel Mechanical Properties Measurement

An aqueous solution (600 µL/well) containing 3.0 *w*/*v*% Gelatin–Ph, 0.5 *w*/*v*% HA–Ph, and 1 U/mL HRP was poured into a well of 12-well plate. The well-plate was then put into a plastic box (13.5 cm × 9.5 cm × 7 cm) and exposed to air containing H_2_O_2_ for 15, 30, 45, 60, 90, and 120 min. The air containing H_2_O_2_ was prepared by bubbling air into a solution containing 1 M H_2_OConcentration of the air containing H_2_O_2_ was measured at 16 ppm using gas detector (C-16 PortaSense II, Analytical Technology, Inc., Collegeville, PA, USA). The Young’s moduli of the resultant Gelatin–Ph/HA–Ph composite hydrogels were measured using a material tester (EZ-Test, Shimadzu, Kyoto, Japan) by compressing the hydrogels with a probe (diameter, 8 mm) at a compression rate of 6.0 mm/min. The Young’s modulus was calculated from the data obtained in the range 1–10% compression strain (Figure S1).

### 2.5. Cell Adhesion and Cell-Cycle Analysis

Hydrogel sheets were fabricated from PBS solution containing 3.0 *w*/*v*% Gelatin–Ph, 0.5 *w*/*v*% HA–Ph, and 1 U/mL HRP. The solution was added to a 12-well plate at 600 µL/well and exposed to air containing 16 ppm H_2_O_2_ for 15, 60, and 120 min. One milliliter culture medium containing 1 mg/mL catalase was then layered on the fabricated hydrogels overnight to degrade the remaining H_2_O_2_ into H_2_O and OThe cell cycle was synchronized to S-phase by incubating the cells in medium containing 4 mM hydroxyurea for 24 h [48]. After the synchronization, cells were trypsinized and seeded on the fabricated gel sheet at 1.0 × 10^4^ cells/well (2.6 × 10^3^ cells/cm^2^). The cell cycle was observed at 1 and 2 days after seeding based on the fluorescence expression of the mCherry-hCdt1 (red) and mVenus-hGem (green) of the Fucci2, using fluorescence microscope (BZ-9000, Keyence, Tokyo, Japan). Red fluorescence indicates G1, yellow indicates G1/S, and green indicates late S/G2/M phase (Figure S2). The number of cells on each phase was counted using ImageJ (2.1.0/1.53c, NIH, Bethesda, MD, USA) from 5–10 randomly selected areas. For live imaging, cells were observed using confocal laser-scanning microscope (CLSM, C2; Nikon, Tokyo, Japan). The 12-well plate containing cells on fabricated hydrogel were placed in a stage-top incubator (STXG-WSKMX; Tokai Hit, Shizuoka, Japan) set at 37 °C supplied with 5% CO_2_ + 20% O_2_ + NTime-lapses were conducted for 48 h. Cell adhesion analysis was conducted by analyzing the morphology of the cells including cell area, perimeter, aspect ratio, and circularity of more than 50 cells for each set of conditions using ImageJ. Aspect ratio was defined as the ratio between major axis/minor axis of the fitted ellipse. Circularity was calculated using the formula: 4π (area/perimeter^2^). Higher aspect ratio indicates elongated morphology. Higher circularity value (near 1.0) indicates a circular shape.

### 2.6. Viability Analysis

Viability of the cells was analyzed by staining the cells with Calcein–AM (Nacalai Tesque Inc., Kyoto, Japan) and propidium iodide (PI, Cellstain^®^, Dojindo, Kumamoto, Japan). Cells were incubated with PBS solution containing 3.3 µg/mL Calcein–AM and PI for 10 min at 37 °C. Live cells stained with Calcein–AM and dead cells stained with PI were observed using fluorescence microscope. Number of viable and dead cells were counted using ImageJ from micrographs taken on 6 randomly selected areas. Viability was calculated as percentage of number of live cells/total number of cells.

### 2.7. F-Actin Analysis

F-actin of the cells was stained with CytoPainter Phalloidin–iFluor 488 Reagent (ab176753, Abcam, Cambridge, UK). Briefly, cells were fixed with 4% paraformaldehyde (PFA) in PBS, permeabilized in HEPES (4-(2-hydroxyethyl)-1-piperazineethanesulfonic acid)-buffered solution (pH 5.5), and stained with CytoPainter Phalloidin–iFluor 488 Reagent (1X) according to the manufacturer’s protocol. The number of cells with stress fibers was counted using ImageJ based on the presence/absence of visible stress fibers (Figure S3). F-actin fluorescence intensity was measured using ImageJ by measuring integrated density defined as sums of all the intensity in the pixel of the selected areas.

### 2.8. Statistical Analysis

Data were statistically analyzed using Microsoft^®^ Excel^®^ 2019 (Version 1808, Microsoft Corp., Redmond, WA, USA). Statistical analysis was conducted using one-way analysis of variance (ANOVA) followed by post hoc *t*-test using Tukey HSD. Data were considered significantly different if *p* < 0.05.

## 3. Results

### 3.1. Mechanical Properties

**Figure 2:**
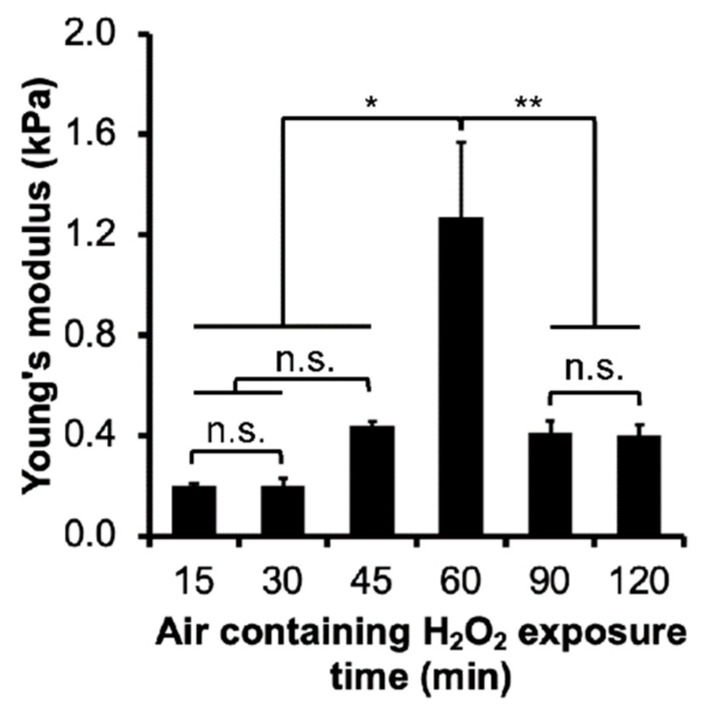
Effect of exposure time to H_2_O_2_ on the Young’s modulus of Gelatin–Ph/HA–Ph composite hydrogels obtained from 3.0 *w*/*v*% Gelatin–Ph, 0.5 *w*/*v*% HA–Ph, and 1 U/mL HRP. Values: Mean ± SE (*n* = 3). * *p* < 0.05, ** *p* < 0.005, n.s.: no significant difference (*p* > 0.05), Tukey HSD.

First, the effect of H_2_O_2_ exposure time (15–120 min) on the stiffness of Gelatin–Ph/HA–Ph composite hydrogels was investigated by measuring the Young’s modulus. Using this setup, hydrogel could be fabricated within 40 s (Figure S4). The result obtained from this experiment showed that H_2_O_2_ exposure time governed the Young’s modulus of the composite hydrogels (*p* < 0.005) (Figure 2). The Young’s modulus of the hydrogels increased as the exposure time to H_2_O_2_ was extended to 45 min, from 0.20 ± 0.01 kPa and 0.20 ± 0.03 kPa at 15 and 30 min of the exposure, respectively, to 0.44 ± 0.02 kPa at 45 min of the exposure. Extending the exposure time to 60 min resulted in the hydrogel with the highest Young’s modulus of 1.27 ± 0.30 kPa. Prolonging the exposure time to 90 and 120 min, however, resulted in the decrease of Young’s modulus to 0.41 ± 0.05 kPa and 0.40 ± 0.04 kPa, respectively (Figure 2).

### 3.2. HeLa/Fucci2 Adhesion

Next, the effect of the exposure time to H_2_O_2_ (15, 60, and 120 min) on the adhesion of the HeLa/Fucci2 cells on the resultant composite hydrogels was investigated. The content of Gelatin–Ph (3.0 *w*/*v*%) and HA–Ph (0.5 *w*/*v*%) in the composite hydrogels used in this study has been reported to allow the elongation of hASCs and support the growth of the cells [49]. As for the exposure time, 15, 60, and 120 min were selected since the Young’s moduli of the composite hydrogels obtained through 15 min (0.20 kPa) and 120 min (0.40 kPa) of H_2_O_2_ exposure were relatively similar. Meanwhile, hydrogel obtained through 60 min of the exposure had the highest Young’s modulus of 1.27 kPa (Figure 2).

**Figure 3:**
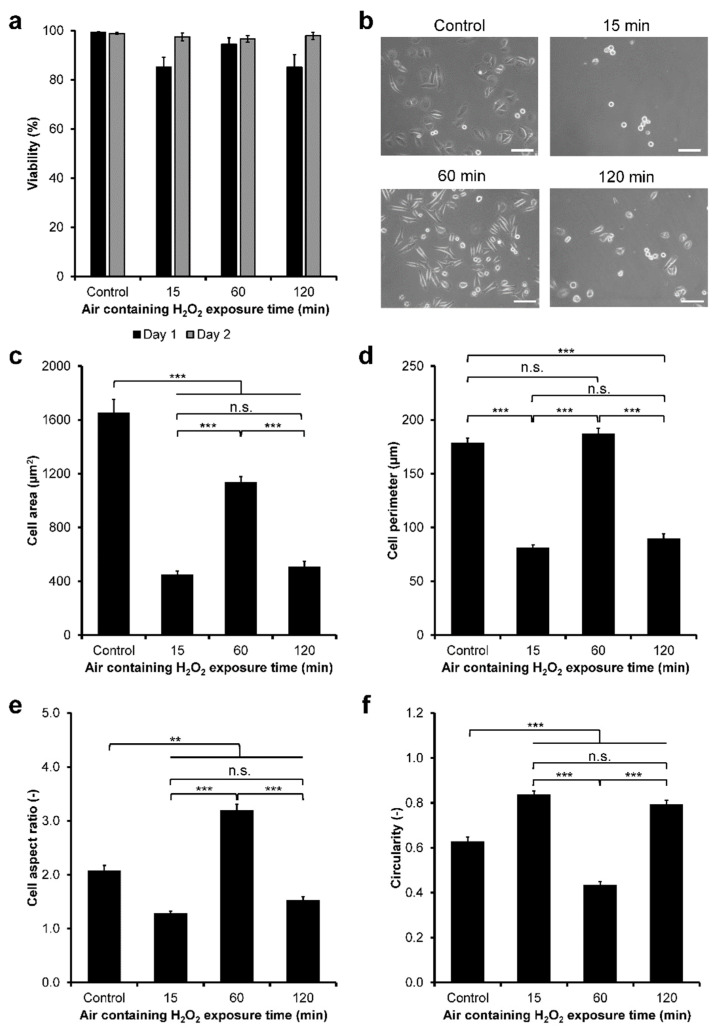
Viability and morphology of HeLa/Fucci2 cell on Gelatin–Ph/HA–Ph composite hydrogels obtained through different exposure time to H_2_O_2_. (**a**) Viability of HeLa/Fucci2 cells calculated based on Calcein–AM/PI staining. Bar: S.E. (*n* = 6) (**b**) Micrograph of HeLa/Fucci2 cells on the composite hydrogels after 1 day cultured on well-plate (control) and the hydrogels obtained through 15, 60, and 120 min exposure to H_2_OScale bars: 100 µm. (**c**) Area, (**d**) perimeter, (**e**) aspect ratio, and (**f**) circularity of HeLa/Fucci2 cells. Bar: S.E. (*n* > 100 cells). ** *p* < 0.005, *** *p* < 0.0005, n.s.: no significant difference (*p* > 0.05) based on Tukey HSD.

First, the viability of the HeLa/Fucci2 cells cultured on the hydrogel was investigated. Viability of the cells was determined by staining the cells with Calcein–AM (green) and PI (red) that stained live and dead cells, respectively (Figure S5). Viability analysis showed that HeLa/Fucci2 cells had high viability (≥85%) from day 1 to day 2 of culture on the hydrogel, independent of the H_2_O_2_ exposure time (Figure 3a).

Next, HeLa/Fucci2 cell adhesion was investigated by analyzing the morphology of the cells. HeLa/Fucci2 cells had different morphology on the hydrogels (Figure 3b). The cells cultured on the hydrogels obtained through 15 min and 120 min exposure to H_2_O_2_ showed no significant difference (*p* > 0.05) in the cell area (Figure 3c), perimeter (Figure 3d), aspect ratio (Figure 3e), and circularity (Figure 3f). These cells appeared to be small and circular indicated by the smallest area and perimeter, lowest aspect ratio, as well as the highest circularity. Meanwhile, the cells cultured on the hydrogel obtained through 60 min of exposure to H_2_O_2_ showed the larger area and perimeter, highest aspect ratio, and the lowest circularity, indicating large and elongated morphology, relatively similar to those on cell culture dish (control, Figure 3b–f).

### 3.3. HeLa/Fucci2 Cell-Cycle Progression

**Figure 4:**
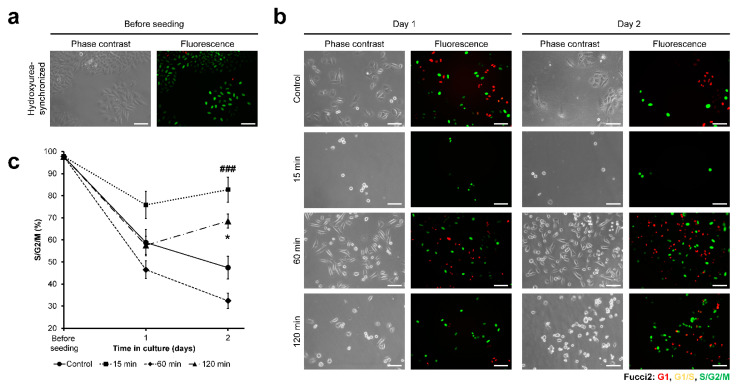
Exposure time to H_2_O_2_-dependent changes in Gelatin–Ph/HA–Ph composite hydrogel stiffness could modulate the cancer cell-cycle progression. (**a**) Phase-contrast and fluorescence observation of the HeLa/Fucci2 before seeding, showing cell synchronized in S-phase, and (**b**) cells at day 1 to day 2 after seeding on the hydrogels. Cells cultured on well-plate were used as control. Fluorescence expression of Fucci2: red indicates G1, yellow indicates G1/S, and green indicates late S/G2/M phase. Scale bar: 100 µm. (**c**) Percentage of HeLa/Fucci2 cells in S/G2/M phase. Bar: S.E. (*n* = 10). ^###^ *p* < 0.0005, 15 min compared to control; * *p* < 0.05, 120 min compared to control, using Tukey HSD.

Next, the cell-cycle progression of HeLa/Fucci2 cells was investigated. Fucci2 allows observation of the expression of mCherry-hCdt1 (red) and mVenus-hGem (green), observed at G1 and S/G2/M, respectively. G1/S transition could be observed as yellow fluorescence (Figure S2). Before seeding, the cell cycle was synchronized with hydroxyurea. After 24 h of 4 mM hydroxyurea exposure, HeLa/Fucci2 predominantly found in S-phase, shown as green fluorescence (Figure 4a). The cells were then seeded on the composite hydrogels obtained through the different time of exposure to H_2_O_2_, and the cell cycle was observed for two days after seeding (Figure 4b).

Cell-cycle analysis shows that the cells on cell culture well-plate (control) showed progression of the cell cycle marked by a continuous decrease in the percentage of cells at S/G2/M phase, from 97.8% before seeding to 58.8% at day 1 and 47.4% at day 2 (Figure 4c). The cells cultured on the hydrogel obtained through 60 min of exposure to H_2_O_2_ showed a similar trend as those on cell well-plate (control, *p* > 0.05), with a decreasing percentage of cells at S/G2/M phase, to 32.4% at day 2 after seeding. In contrast, the cells cultured on the hydrogels obtained through 15 and 120 min of the exposure remained at S/G2/M phase, with 82.7% and 68.5% of the cells were found in S/G2/M phase at day 2, respectively (Figure 4c). To further confirm this observation, a live-imaging experiment was conducted by observing the HeLa/Fucci2 cells from 8–48 h post-seeding. Live-imaging observation showed higher mitosis frequency in HeLa/Fucci2 cells cultured on 60 min composite hydrogel and control (Movie S1). Additionally, cell growth was analyzed by calculating the cell density on day 1 and day 2 post-seeding. Cells cultured on hydrogel obtained from 15 and 120 min of the exposure showed no significant growth in cells density from day 1 to day 2 (*p* > 0.05). On the other hand, cells cultured on hydrogel obtained from 60 min exposure time to H_2_O_2_ and control cultured on well-plate showed a significant increase in cell density (*p* < 0.05) (Figure S7).

### 3.4. HeLa/Fucci2 F-Actin Reorganization

**Figure 5:**
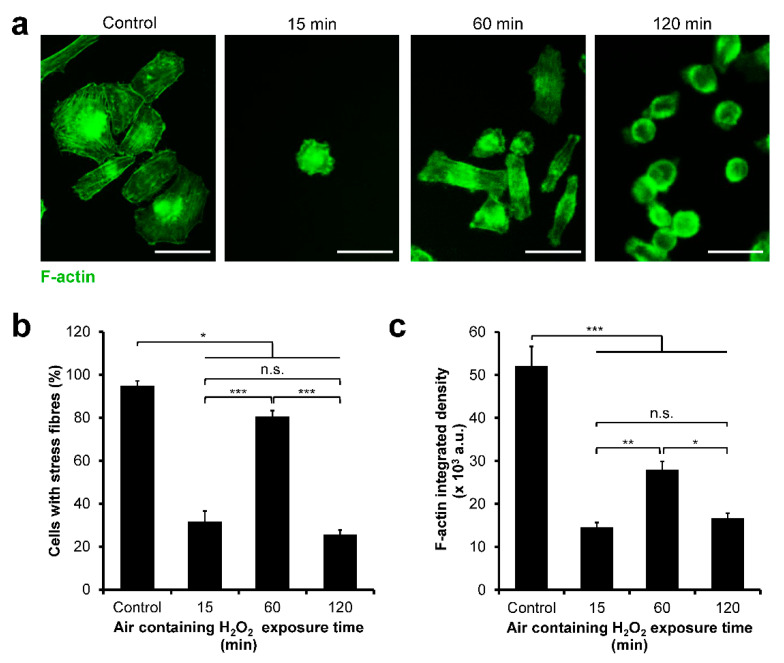
F-actin reorganization in HeLa/Fucci2 cells cultured on Gelatin–Ph/HA–Ph composite hydrogels obtained through 15, 60, and 120 min of exposure to H_2_O_2_. (**a**) Fluorescence observation of the F-actin of HeLa/Fucci2 cells cultured on well-plate (control) and the hydrogels. Scale bar: 100 µm. (**b**) Percentage of cells with stress fibers. Bar: S.E. (*n* = 6). (**c**) F-actin integrated density. Bar: S.E. (*n* = 45 cells). * *p* < 0.05, ** *p* < 0.005, *** *p* < 0.0005, n.s.: no significant difference, Tukey HSD.

The difference in cell-cycle progression of the cells cultured on each hydrogel might be mediated by the cytoskeleton reorganization. Therefore, the F-actin of the cells was stained, and the formation of stress fibers was investigated (Figure 5a). Most of the cells cultured on cell culture well-plate (control) and those on the hydrogel obtained through 60 min of exposure to H_2_O_2_ had stress fibers. However, cells cultured on the hydrogel obtained through 15 and 120 min of the exposure had a significantly lower percentage of cells with stress fibers (*p* < 0.0005) (Figure 5b). A similar trend was observed in F-actin integrated density with cells cultured on the hydrogel obtained through the 60 min of exposure to H_2_O_2_: the cells had significantly higher integrated density (*p* < 0.05) compared to those on the hydrogel obtained through 15 and 120 min of the exposure to H_2_O_2_ (Figure 5c).

### 3.5. NMuMG/Fucci2 Adhesion

**Figure 6:**
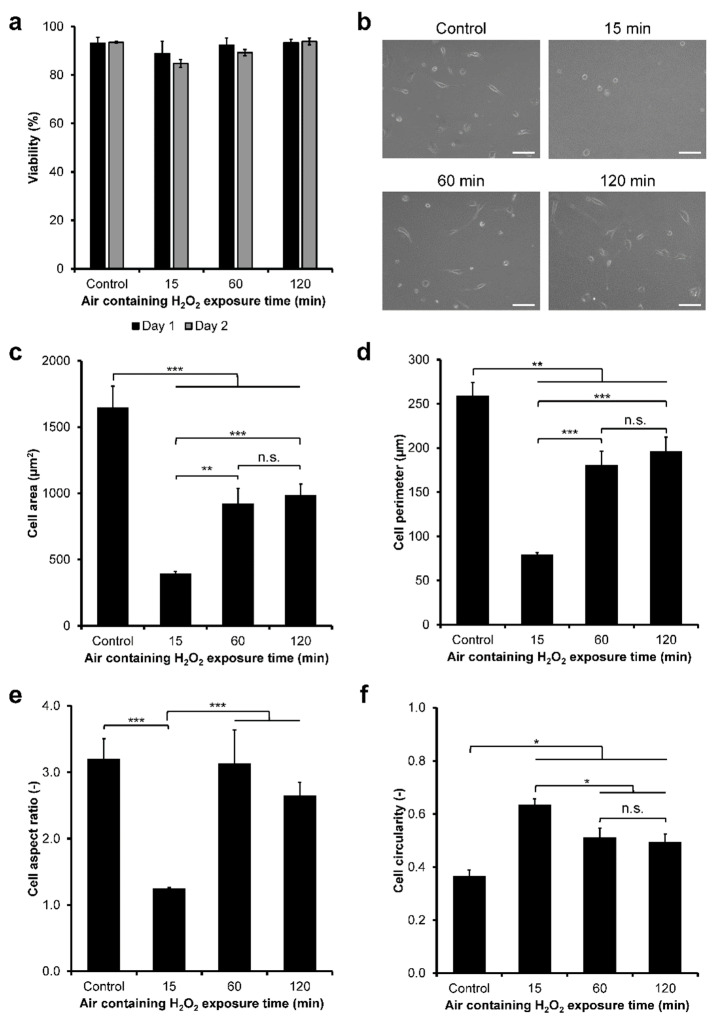
Viability and morphology of NMuMG/Fucci2 cells on Gelatin–Ph/HA–Ph hydrogel obtained through different exposure time to H_2_O_2_ in the air. (**a**) Viability of NMuMG/Fucci2 cells on day 1 and day 2 of culture. Bar: S.E. (*n* = 6). (**b**) Representative phase-contrast image of NMuMG/Fucci2 cells on the hydrogels obtained through 15, 60, and 120 min of the exposure. As a control, cells were cultured on plastic surface of the well-plate. Scale bars: 100 µm. (**c**) Area, (**d**) perimeter, (**e**) aspect ratio, and (**f**) circularity of NMuMG/Fucci2 cells. Bar: S.E. (*n* > 50 cells). * *p* < 0.05, ** *p* < 0.005, *** *p* < 0.0005, n.s.: no significant difference, Tukey HSD.

Next, the behaviors of NMuMG/Fucci2 cells on the composite hydrogels were investigated. First, the viability of NMuMG/Fucci2 cells on the composite hydrogels was investigated by staining the cells with Calcein–AM/PI (Figure S6). The viability of those NMuMG/Fucci2 cells cultured on the Gelatin–Ph/HA–Ph composite hydrogel was ≥84% (Figure 6a). Additionally, air containing H_2_O_2_ exposure time did not induce significant changes (*p* > 0.05) in the viability of the NMuMG/Fucci2 cells.

The morphology of the NMuMG/Fucci2 cells on the composite hydrogel was then investigated. The cells on plastic surface of the well-plate (control) and those on the hydrogels obtained through 60 and 120 min of exposure to H_2_O_2_ elongated similarly (Figure 6b). The cells on the hydrogel obtained through 15 min of the exposure showed a small and round morphology attributed to the smallest area (Figure 6c) and perimeter (Figure 6d), aspect ratio near 1.0 (Figure 6e), and highest circularity (Figure 6f). The cells on the hydrogel obtained through 60 min of the exposure showed significantly larger and elongated shapes attributed to a larger area and perimeter, higher aspect ratio, and lower circularity, compared to cells on hydrogel obtained through 15 min of the exposure. Interestingly, the cells on the hydrogel obtained through 120 min of the exposure had no significant differences (*p* > 0.05) in their morphological characteristics as to those cultured on the hydrogel obtained through 60 min of the exposure.

### 3.6. NMuMG/Fucci2 Cell-Cycle Progression

**Figure 7:**
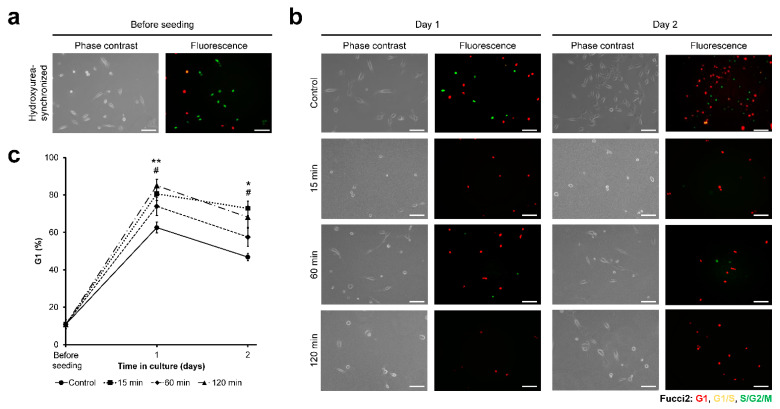
NMuMG/Fucci2 cell-cycle progression on Gelatin–Ph/HA–Ph composite hydrogels obtained through different exposure time to H_2_O_2_. (**a**) Representative micrograph of NMuMG/Fucci2 cells before seeding, synchronized by hydroxyurea to S-phase, and (**b**) NMuMG/Fucci2 cells on Gelatin–Ph/HA–Ph hydrogel and well-plate (control) for 2 days. Scale bars: 100 µm. (**c**) Percentage of NMuMG/Fucci2 cells in G1 phase. Bar: S.E. (*n* ≥ 5). ^#^ *p* < 0.05, 15 min compared to control; * *p* < 0.05, ** *p* < 0.005, 120 min compared to control, using Tukey HSD.

Next, the cell-cycle progression of NMuMG/Fucci2 cells on the composite hydrogels was investigated. Hydroxyurea synchronization before seeding resulted in 84.0% S-phase cells (Figure 7a). Cell-cycle observation based on the Fucci2 expression (Figure 7b) showed that the cells on the hydrogels obtained through 15 and 120 min of exposure to H_2_O_2_ had a significantly higher (*p* < 0.05) percentage of cells at G1 phase from day 1 to day 2 on the hydrogels compared to those on cell well-plate (control) (Figure 7c). In contrast, the percentage of G1-phase cells on the hydrogel obtained through 60 min of the exposure was not significantly different from that of control (*p* > 0.05) (Figure 7c). Time-lapse observation also showed higher mitotic cells in control and those cultured on composite hydrogel obtained from 60 min H_2_O_2_ exposure time, compared to cells on 15 min and 120 min hydrogel (Movie S2). Cell growth analysis also showed significant cell density increase only in cells cultured on well-plate (control) and 60 min hydrogel (Figure S8).

### 3.7. NMuMG/Fucci2 F-Actin Reorganization

**Figure 8:**
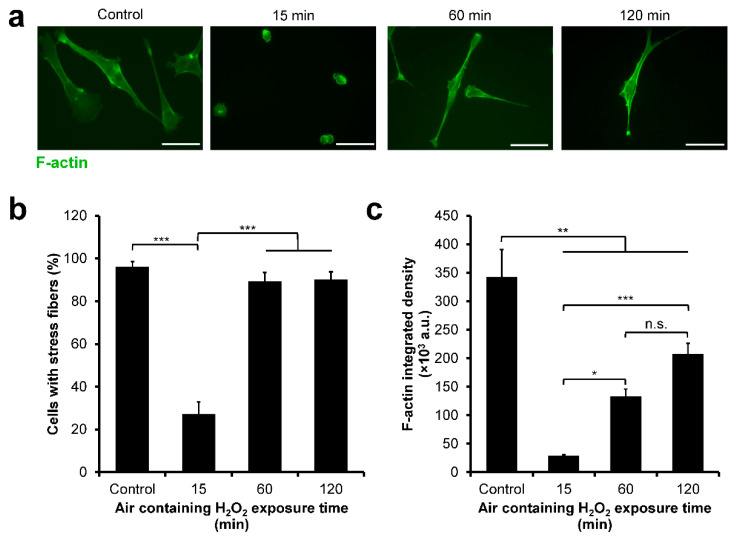
F-actin reorganization of NMuMG/Fucci2 cells cultured on Gelatin–Ph/HA–Ph composite hydrogel obtained through different exposure time to H_2_O_2_ in the air. (**a**) Representative fluorescence micrograph of the F-actin of NMuMG/Fucci2 cells cultured on Gelatin–Ph/HA–Ph hydrogel obtained through 15–120 min of exposure to H_2_OCells cultured on well-plate were used as control. Scale bars: 50 µm. (**b**) Comparison of percentage of cells with stress fibers. Bar: S.E. (*n* ≥ 5). (**c**) F-actin integrated density of NMuMG/Fucci2 cells. Bar: S.E. (*n* = 50 cells). * *p* < 0.05, ** *p* < 0.005, *** *p* < 0.0005, n.s.: no significant difference based on Tukey HSD.

Next, the F-actin organization of the NMuMG/Fucci2 cells was investigated by staining the actin stress fibers of the cells (Figure 8a). Analysis of the presence of stress fibers showed that the cells cultured on the composite hydrogels obtained through 15 min of exposure to H_2_O_2_ had the lowest percentage of cells with stress fibers (Figure 8b). Similarly, these cells had the lowest F-actin integrated density (Figure 8c). In contrast, 89.4% of the cells on the hydrogel obtained through 60 min of the exposure had stress fibers, relatively similar to those on cell culture well-plate (control, 96.1%) (Figure 8b) and significantly higher F-actin integrated density (*p* < 0.05) than those on the hydrogel obtained through 15 min of the exposure (Figure 8c). Interestingly, the cells cultured on the hydrogel obtained through 120 min of the exposure had no significant difference (*p* > 0.05) in the presence of actin stress fibers (Figure 8b) and F-actin integrated density (Figure 8c) compared to the cells cultured on the hydrogel obtained through 60 min of exposure to H_2_O_2_.

## 4. Discussion

In this study, we aimed to investigate the effect of the contradictory effect of H_2_O_2_ on the stiffness of Gelatin–Ph/HA–Ph composite hydrogel and its subsequent effect on the modulations of the cell adhesion and cell-cycle progression. Cell-cycle regulation is an important process, particularly with regards to cancer development. Most studies have used anticancer drugs to study the cell-cycle regulation of cancer cells [50,51]. However, few studies have investigated the cancer cell-cycle regulation by the physicochemical properties of their ECM. Here, we used the composite hydrogel to mimic the native ECM environment, which provided a better model for the phenomenon in the native tissues and study for tissue-engineering applications. In this cross-linking system, we used H_2_O_2_ exposure time to control the mechanical property of the Gelatin–Ph/HA–Ph hydrogels obtained through HRP-mediated cross-linking.

The mechanism of the exposure time-dependent transition of the stiffness of the hydrogels can be explained by the correlation between the degrees of the progress of cross-linking formation catalyzed by HRP, HRP inactivation, and Gelatin–Ph/HA–Ph degradation, which all involve H_2_OThe inactivation of HRP [52,53] and degradation of organic molecules including Gelatin and HA by H_2_O_2_ has been reported [23,54]. In this study, H_2_O_2_ was supplied continuously to the system containing Gelatin–Ph/HA–Ph and HRP. Thus, the content of the cross-linked Ph moieties should increase by extending the exposure time to H_2_O_2_, unless HRP inactivation and Gelatin–Ph/HA–Ph degradation occurred.

Therefore, the results shown in Figure 2 that demonstrate the decrease of Young’s modulus with increasing the exposure time from 60 to 120 min, indicate the occurrences of Gelatin–Ph/HA–Ph degradation. Indeed, we confirmed that H_2_O_2_ could degrade the Gelatin–Ph and HA–Ph polymer, based on the molecular weight measurement (Figure S9). Taken together, a tunable control of Gelatin–Ph/HA–Ph Young’s modulus was achieved by simple control of the exposure time to H_2_O_2_ contained in the air.

Next, we investigated the adhesion and cell-cycle progression of cervical cancer cells using HeLa/Fucci2 cells. Modulation of the cell cycle of cancer cells has garnered great interest to study the progression of tumors and inhibition of tumor growth [1,2]. Changes in the physicochemical properties in ECM as well as cell–ECM adhesion are also known to modulate tumor cells invasion and metastasis [55,56]. First, we investigated the viability of the cells. HeLa/Fucci2 cells showed high viability regardless of the air containing H_2_O_2_ exposure time (Figure 3a; Figure S5). This result could be attributed to the well-known biocompatibility of both Gelatin–Ph and HA–Ph [49,57,58]. Next, our results showed that HeLa/Fucci2 cells had mechanical property-dependent changes in cell adhesion (Figure 3b–f) and cell-cycle progression (Figure 4). HeLa/Fucci2 cells cultured on soft hydrogel obtained through 15 min (0.20 kPa) and 120 min (0.40 kPa) of H_2_O_2_ exposure had similar morphology with small size and round shape. Meanwhile, HeLa/Fucci2 cells cultured on stiffer hydrogel (1.27 kPa) obtained through 60 min of the exposure had larger size and elongated morphology (Figure 3b–f). The stiffness-dependent change in cell morphology is consistent with the results of previous studies using hASCs cultured in Gelatin–Ph/HA–Ph hydrogel [49] and fibroblast cells on DNA cross-linked hydrogel, which showed a spherical shape on soft substrates and an elongated shape on stiff substrates [59]. Based on these results, H_2_O_2_ exposure time-mediated changes to Gelatin–Ph/HA–Ph stiffness could govern the adhesion of HeLa/Fucci2 cells.

As for the cell-cycle progression, we found that stiffer hydrogel is required for HeLa/Fucci2 cell-cycle progression. Cells cultured on soft hydrogel obtained through 15 min and 120 min of exposure to H_2_O_2_ had an arrested cell cycle at G2/M phase (Figure 4), which resulted in halted cell growth (Figure S7). In contrast, cells on the hydrogel obtained through 60 min of H_2_O_2_-exposure had similar progression to those of control (Figure 4), which allows cell growth (Figure S7). Our result is consistent with the results reported by Paszek et al. [60]. They reported that cancer cells cultured on soft collagen gel had lower growth and adhesion [60]. Wall et al. reported that melanoma cells cultured in fibrillar collagen had a cell-cycle arrest in G2 [61]. The progression of the cell cycle on stiffer substrates observed in this study is consistent with the results reported by Klein et al. about the cell-cycle progression in stiff polyacrylamide hydrogels, but not in soft hydrogels, after nocodazole-mediated G2/M arrest [62].

The difference in the cell-cycle progression of cells cultured on soft and stiff hydrogel could be attributed to the F-actin assembly. Previous studies have reported that actin plays a key role in mitosis [63,64]. Indeed, we found that HeLa/Fucci2 cells had lower F-actin assembly on soft hydrogel obtained through 15 min (0.20 kPa) and 120 min (0.40 kPa) of H_2_O_2_ exposure (Figure 5). Previous studies have reported that actin disruption could lead to G2/M arrest in transformed cancer cell line IMR-90 and MCF-7 cells [65,66]. Accordingly, cells cultured on composite hydrogel obtained through 15 min and 120 min of H_2_O_2_ exposure that showed a reduction in stress fibers assembly (Figure 5) also showed G2/M arrest (Figure 4). This indicates that one of the possible causes of cell-cycle arrest at G2/M phase observed in the HeLa/Fucci2 cells cultured on soft hydrogel obtained through 15 min and 120 min of exposure to H_2_O_2_ is due to the reduction in stress fibers assembly.

Next, the adhesion and cell-cycle progression of NMuMG/Fucci2 cells was investigated. Similar to the result in HeLa/Fucci2 cells, NMuMG/Fucci2 cells showed high viability from day 1 to day 2 on the Gelatin–Ph/HA–Ph composite hydrogel, independent of the H_2_O_2_ exposure time (Figure 6a; Figure S6). Interestingly, we found some responses in NMuMG/Fucci2 cells that differ from HeLa/Fucci2 cells. Cell morphology analysis showed that while NMuMG/Fucci2 cells also had small and round morphology on the hydrogel obtained through 15 min of exposure to H_2_O_2_, cells cultured on the hydrogel obtained through 120 min of the exposure had an elongated shape, despite having almost similar stiffness (0.40 kPa) with hydrogel obtained through 15 min of the exposure (0.20 kPa) (Figure 6b–f). This phenomenon is similar to our previous report in human adipose-derived stem cells (hASCs) and rat fibroblast (3Y1) cells cultured on Gelatin–Ph hydrogel that showed elongated morphology on the hydrogel obtained through 60 min of exposure to H_2_O_2_ despite having similar stiffness with hydrogel obtained through 15 min of the exposure [31]. This phenomenon could be attributed to other factors such as mesoscale topographical changes and mesh size [67]. In the prolonged exposure time to H_2_O_2_, the phase-separation phenomenon might occur, resulting in the formation of a domain. Phase separation could be observed from the cloudy appearance of the fabricated hydrogel (Figure S10). This phase separation could induce clustering of cell surface receptors (see [68] for review), which could induce alteration of cell shape. Another factor that could induce better adhesion in the cells cultured on the hydrogel obtained through 120 min of the exposure is the low-molecular-weight fragments out of the polymer. As shown in Figure S9, H_2_O_2_ could degrade both Gelatin–Ph and HA–Ph. HA is well-known to have different modulatory effects based on their molecular weight [17,18,19]. Low-molecular-weight HA (LMW-HA) is reported to induce better adhesion in human epithelial cells HT1080, mediated by receptor for hyaluronic acid-mediated motility (RHAMM) [69]. LMW-HA also induces EGF-induced branching of mammary epithelial cells EpH4 and NMuMG cells, while the high-molecular-weight HA inhibits the morphological changes, a process that is mediated by HA receptors CD44 and RHAMM [70].

Another difference was found in the cell-cycle progression. Although HeLa/Fucci2 cells showed cell-cycle arrest at G2/M (Figure 4) in soft hydrogel obtained 15 min (0.20 kPa) and 120 min (0.40 kPa) of H_2_O_2_ exposure, NMuMG/Fucci2 cells showed an arrested cell-cycle at G1 phase (Figure 7). This difference was also reported in the previous report by Sakaue-Sawano et al. (2011), in which the cell type-dependent response is observed following exposure to Cdk4 inhibitor drugs, with HeLa/Fucci2 cells arrested at G2/M phase while NMuMG/Fucci2 cells are arrested at G1 phase [43]. At the moment, the intricate mechanism on how the H_2_O_2_ exposure time to Gelatin–Ph/HA–Ph hydrogel induced cell-cycle arrest at a different phase in HeLa/Fucci2 and NMuMG/Fucci2 is unknown. However, there is a possibility that NMuMG/Fucci2 cultured on Gelatin–Ph/HA–Ph hydrogel obtained through 120 min of the exposure had an epithelial-to-mesenchymal transition (EMT).

EMT is a physiological transition or reprogramming of epithelial cells to mesenchymal cells, marked by a morphological change into spindle-like mesenchymal cells, which plays an important role in the malignancy and invasiveness of cancer [44,45]. Recent study by Fan et al. (2021) shows that soft substrate (0.5 kPa) promotes the EMT in SKOV-3 ovarian cancer cells [71]. This stiffness is close with the Young’s modulus of our Gelatin–Ph/HA–Ph hydrogel obtained through 120 min of H_2_O_2_-exposure at 0.40 kPa. Moreover, LMW-HA is also known to induce the EMT in MCF-7 and MDA-MB-231 cells [72]. This might explain why despite both Gelatin–Ph/HA–Ph composite hydrogel obtained through 15 min (0.20 kPa) and 120 min (0.40 kPa) of H_2_O_2_ exposure had low Young’s modulus, but, the NMuMG/Fucci2 cells adhesion (Figure 6) and F-actin organization (Figure 8) were different: the molecular weight of HA–Ph exposed to H_2_O_2_ for 120 min was probably lower due to H_2_O_2_ degradation, which promotes EMT due to interaction with HA receptors such as CD44 or RHAMM.

As for the cell-cycle arrest in G1 observed in NMuMG/Fucci2 cells cultured on the hydrogel obtained through 120 min of H_2_O_2_ exposure (Figure 7), despite having similar F-actin organization with cells cultured on the hydrogel obtained through 60 min of H_2_O_2_ exposure (Figure 8), this could also be explained by the EMT process. EMT is known to induce cell-cycle arrest [73,74,75]. Additionally, *Snai1* and *Snai2* (Slug) which belong to the SNAIL family transcription factors that regulate the EMT process are also known to regulate G1 phase transition [76].

Taken together, the contradictory effect of H_2_O_2_ to induce HRP-mediated cross-linking and degrade the Gelatin–Ph and HA–Ph polymer could affect the adhesion and cell-cycle progression of HeLa/Fucci2 and NMuMG/Fucci2 cells in cell type-dependent manner. These findings can be useful in biomedical field especially for creating tissues by combining cells and hydrogels obtained through HRP-mediated cross-linking. Development of various artificial tissues have been attempted by combining cells and the hydrogels for surgical transplantation and in vitro model for drug screening [77,78]. The control and understanding of the cell cycle in these engineered tissues are important for creating functional tissues and for accurately interpreting cellular responses to drug candidates, including anticancer drugs. Additionally, phenomenon reported in our study could be used to study the cancer cell behavior affected by the physicochemical property of their surrounding ECM. Future studies should be conducted to confirm the molecular mechanism of the cell-cycle regulation, adhesion, and also possibly EMT, by the mechanical property of the hydrogel and molecular weight of the polymer. Additionally, the adhesion and cell-cycle regulation of primary cancer cells on the composite hydrogel also needs to be investigated.

## 5. Conclusions

In this study, we investigated the H_2_O_2_ effect to induce HRP-mediated cross-linking and degrade polymer in Gelatin–Ph/HA–Ph composite hydrogel and its subsequent effect on cell adhesion and cell-cycle progression. Young’s moduli of the Gelatin–Ph/HA–Ph composite hydrogels could be controlled by simple adjustment of the exposure time to H_2_O_2_ contained in the air, in which the stiffness increased after extending the exposure time from 15 to 60 min, followed by a decrease in extended exposure time to 120 min. The exposure time-dependent changes to the hydrogel stiffness governed the adhesion and cell-cycle progression of HeLa/Fucci2 cells and NMuMG/Fucci2 cells, in a cell-type-dependent manner. Although soft hydrogel leads to cell-cycle arrest at G2/M phase for HeLa/Fucci2, NMuMG/Fucci2 cells were arrested at G1 phase. Apart from hydrogel stiffness, the NMuMG/Fucci2 cell-cycle progression was also modulated by the H_2_O_2_-mediated degradation of HA in the composite, which produced low-molecular-weight HA. From these results, we conclude that the contradictory effects of H_2_O_2_ to Gelatin–Ph/HA–Ph composite hydrogel could be used to control the cell adhesion and cell-cycle progression, which could be used in cancer studies and tissue-engineering purposes.

## Figures and Tables

**Table 1:** List of abbreviations. The abbreviations are reported alphabetically.

Abbreviation	Full Form
CLSM	Confocal laser-scanning microscope
DMEM	Dulbecco’s Modified Eagle Medium
DMF	*N*,*N*-dimethylformamide
ECM	Extracellular matrix
EGF	Epidermal growth factor
EMT	Epithelial-to-mesenchymal transition
FBS	Fetal bovine serum
Fucci	Fluorescent ubiquitination-based cell-cycle indicator
HA	Hyaluronic acid
hASCs	Human adipose-derived stem cells
HEPES	4-(2-hydroxyethyl)-1-piperazineethanesulfonic acid
HRP	Horseradish peroxidase
LMW-HA	Low-molecular-weight hyaluronic acid
MW	Molecular weight
NHS	*N*-hydroxysuccinimide
PBS	Phosphate buffered saline
PFA	Paraformaldehyde
Ph	Phenolic hydroxyl moieties
PI	Propidium iodide
RGD	Tripeptide arginine–glycine–aspartic acid
RHAMM	Receptor for hyaluronic acid-mediated motility
WSCD·HCl	Water-soluble carbodiimide hydrochloride

## Data Availability

All data generated or analyzed during this study are included in this published article and its supplementary information files.

## Supplementary Materials

The following supporting information can be downloaded at: https://www.mdpi.com/article/10.3390/cells11050881/s1, Figure S1: Stress–strain curves of Gelatin–Ph/HA–Ph hydrogel obtained from 3.0 *w*/*v*% Gelatin–Ph + 0.5 *w*/*v*% HA–Ph and 1 U/mL HRP exposed with various air containing H_2_O_2_ exposure time; Figure S2: Diagram of the Fucci2 expression and representative confocal laser-scanning microscope observation of the Fucci2 expression; Figure S3: Representative image of cells with stress fibers and no stress fibers; Figure S4: Gelation time of 3.0 *w*/*v*% Gelatin–Ph/0.5 *w*/*v*% HA–Ph solutions containing 1, 5, and 15 U/mL HRP exposed to air containing H_2_O_2_; Figure S5: HeLa/Fucci2 cells stained with Calcein–AM and propidium iodide indicating live and dead cells, respectively; Figure S6: Fluorescence micrograph of live and dead NMuMG/Fucci2 cells stained with Calcein–AM and PI; Figure S7: Density of HeLa/Fucci2 cells on Gelatin–Ph/HA–Ph composite hydrogel obtained from different H_2_O_2_ exposure time; Figure S8: NMuMG/Fucci2 cell density cultured on Gelatin–Ph/HA–Ph hydrogel obtained from varying H_2_O_2_ exposure time; Figure S9: Degradation of Gelatin–Ph and HA–Ph polymer by H_2_O_2_; Figure S10: Phase separation in Gelatin–Ph/HA–Ph composite hydrogel fabricated after exposure to H_2_O_2_ for 15 to 120 min. Movie S1: Live imaging of HeLa/Fucci2 cells on well-plate (control) and Gelatin–Ph/HA–Ph composite hydrogel obtained from 15, 60, and 120 min of H_2_O_2_ exposure time. Live imaging conducted from 8–48 h post-seeding; Movie S2: Time-lapse observation of NMuMG/Fucci2 cells on Gelatin–Ph/HA–Ph hydrogel obtained from different exposure time to air containing H_2_O_2_ from 8–48 h after seeding.
