# Supplementary material for: Modulation of Cell-Cycle Progression by Hydrogen Peroxide-Mediated Cross-Linking and Degradation of Cell-Adhesive Hydrogels
Source: Cells. 2022 Mar 3;11(5):881. doi: 10.3390/cells11050881 (PMC8909037; doi:10.3390/cells11050881)
Supplement: Supplementary file 1 [file cells-11-00881-s001.zip › Supplementary Information.pdf]

cells

MDPI

Supplementary Information

Modulation of Cell-Cycle Progression by Hydrogen Peroxide-Mediated Cross-Linking and Degradation of Cell-Adhesive Hydrogels

Wildan Mubarok, Kelum Chamara Manoj Lakmal Elvitigala, Masaki Nakahata, Masaru Kojima and Shinji Sakai*

Department of Materials Engineering Science, Graduate School of Engineering Science, Osaka University, Toyonaka, Osaka 560-8531, Japan
*Correspondence: sakai.shinji.es@osaka-u.ac.jp; Tel.: +81-6-6850-6252

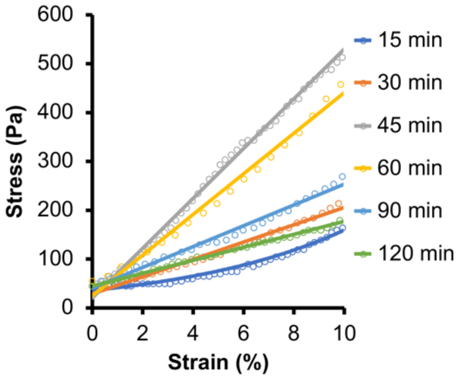

Figure S1. Stress–strain curves of Gelatin–Ph/HA–Ph hydrogel obtained from 3.0 *w*/*v*% Gelatin–Ph + 0.5 *w*/*v*% HA–Ph and 1 U/mL HRP exposed with various air containing H_2O_2 exposure time.

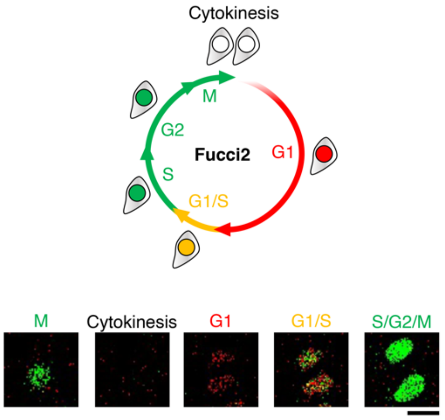

Figure S2. Diagram of the Fucci2 expression and representative confocal laser-scanning microscope observation of the Fucci2 expression. mCherry-hCdt1 is expressed in G1 phase shown as red fluorescence, mVenus-hGem expressed in late S/G2/M phase, and G1/S transition observed as yellow fluorescence (co-expression of red and green). Scale bar: 10 μm.

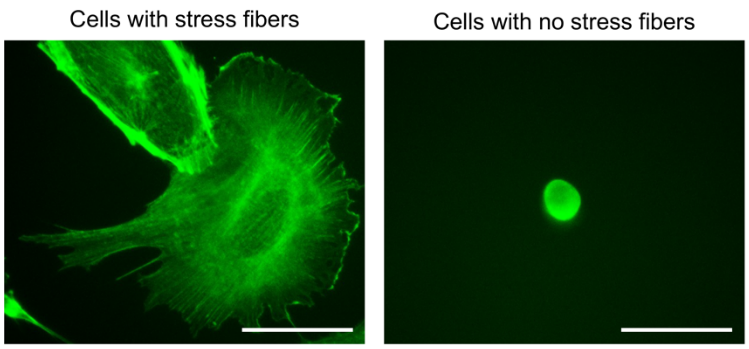

Figure S3. Representative image of cells with stress fibers and no stress fibers. Cells with stress fibers are characterized by the visible fibers in the cell's cytoplasm and nucleus. Cells: NMuMG/Fucci2 cells. Scale bars: 50 µm.

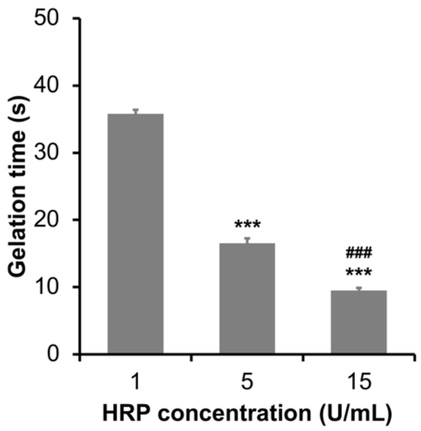

Figure S4. Gelation time of 3.0 *w*/*v*% Gelatin–Ph / 0.5 *w*/*v*% HA–Ph solutions containing 1, 5, and 15 U/mL HRP exposed to air containing H_2O_2. Bar: S.E. (n=5). ***p<0.0005, compared to 1 U/mL, $^{###}p$<0.0005 compared to 5 U/mL, Tukey HSD.

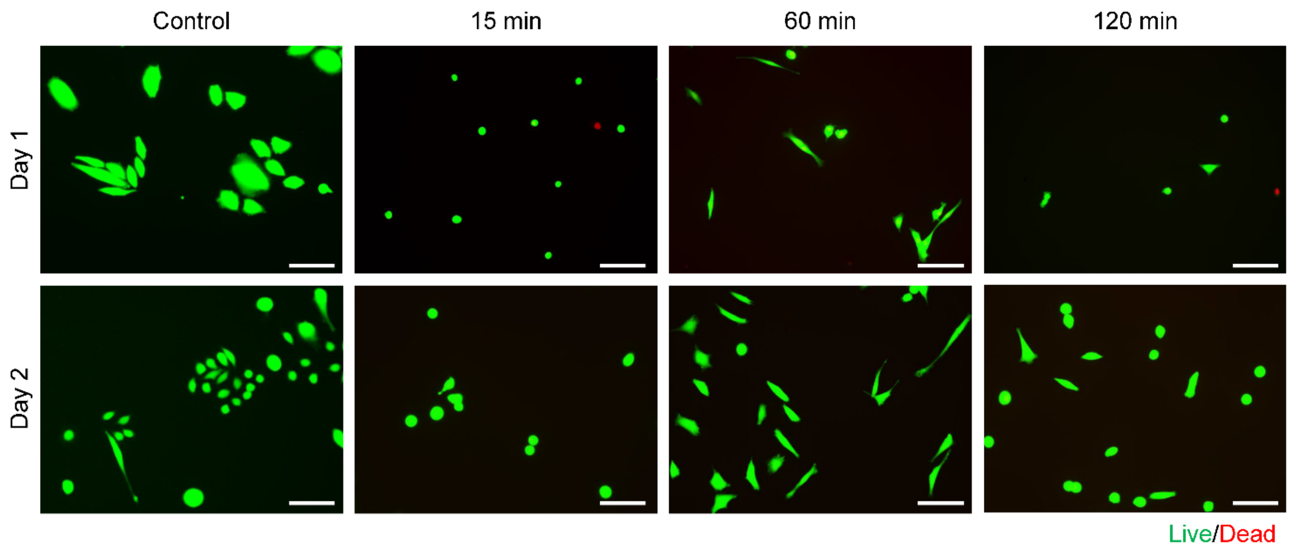

Figure S5. HeLa/Fucci2 cells stained with Calcein–AM (green) and propidium iodide (PI, red) indicating live and dead cells, respectively. Scale bar: 100 µm.

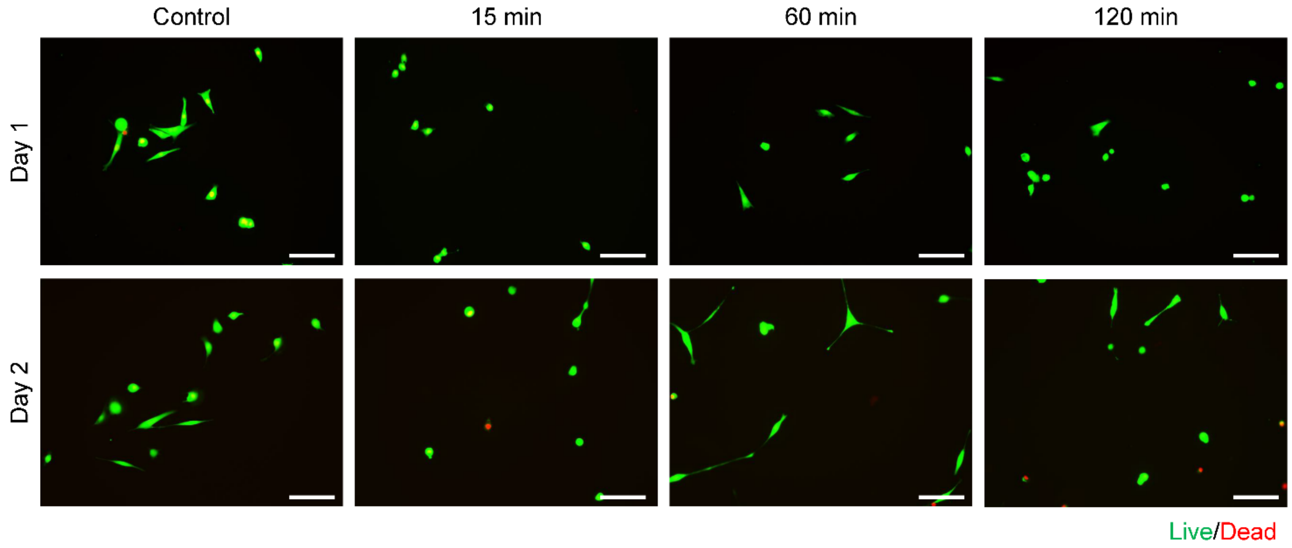

Figure S6. Fluorescence micrograph of live and dead NMuMG/Fucci2 cells stained with Calcein–AM and PI. Scale bar: 100 µm.

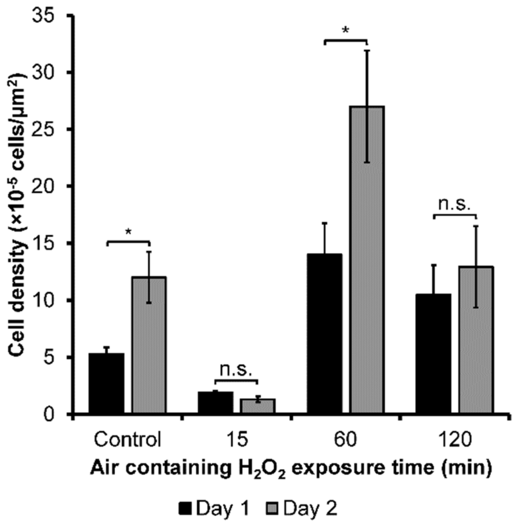

Figure S7. Density of HeLa/Fucci2 cells on Gelatin–Ph/HA–Ph composite hydrogel obtained from different H_2O_2 exposure time. Bar: S.E. (n=5). *p<0.05, n.s.: no significant difference (p>0.05), Tukey HSD.

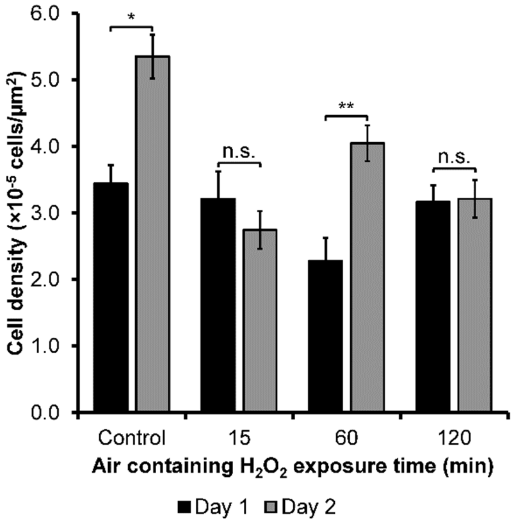

Figure S8. NMuMG/Fucci2 cell density cultured on Gelatin–Ph/HA–Ph hydrogel obtained from varying H_2O_2 exposure time. Bar: S.E. (n=5). *p<0.05, **p<0.005, n.s.: no significant difference, Tukey HSD.

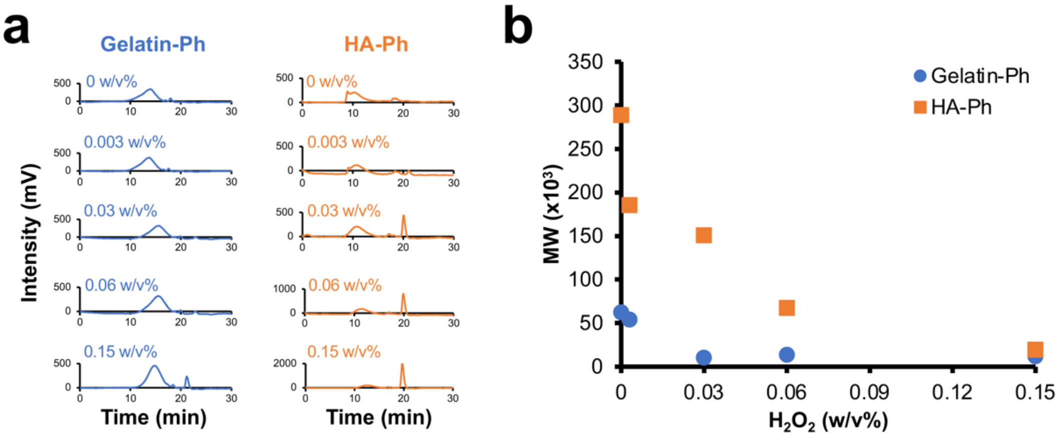

Figure S9. Degradation of Gelatin–Ph and HA–Ph polymer by H_2O_2. (a) Intensity-time curve of 3.0 w/v% Gelatin-Ph and 0.5 w/v% HA-Ph treated by 0%, 0.003%, 0.03%, 0.06% and 0.15% (w/v%) H_2O_2 solution. (b) Peak top molecular weight, relative to the polyethylene glycol (standard), of 3.0 w/v% Gelatin-Ph and 0.5 w/v% HA-Ph after overnight incubation with H_2O_2. LC conditions: separation was performed on a SHIMADZU HPLC system equipped with Shodex OHpak SB-804 HQ, 8.0 mmID x 300 mmL column; mobile phase PBS at pH 7.4; flow rate, 0.7 mL/min; temperature, 25˚C.

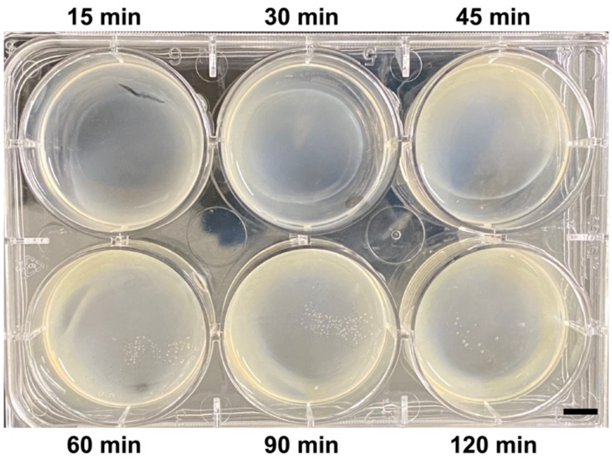

Figure S10. Phase separation in Gelatin–Ph/HA–Ph composite hydrogel fabricated after exposure to H_2O_2 for 15 to 120 min. Notice the cloudy appearance of the hydrogel in extended exposure time, indicating phase-separation occurrence. Scale bar: 5 mm.
